# Oral health status and oral health knowledge, attitudes and behavior among rural children in Shaanxi, western China: a cross-sectional survey

**DOI:** 10.1186/1472-6831-14-144

**Published:** 2014-11-29

**Authors:** Jianghong Gao, Jianping Ruan, Lin Zhao, Hong Zhou, Ruizhe Huang, Jiangang Tian

**Affiliations:** Department of Oral Prevention, School of Stomatology, Xi’an JiaoTong University Health Science Center, #98 Xiwu Road, Xi’an, 710004 Shaanxi Province China; Department of Oral Orthodontics, School of Stomatology, Xi’an JiaoTong University Health Science Center, Xi’an, Shaanxi Province China

**Keywords:** Oral health status, Risk factors, Village children, Western China

## Abstract

**Background:**

The current oral health status and possible dental risk factors among children in rural Shaanxi Province, western China are unreported. This study aimed to describe the oral health status and to analyze the possible risk factors for the oral health status in this population.

**Methods:**

A multi-stage cluster sampling method was used to survey 12- to 15-year-olds and 4- to 6-year-olds in villages in Shaanxi Province. The structured questionnaires were provided to the 12- to 15-year-olds and to the caregivers of the 4- to 6-year-olds to collect information on the subjects’ oral health knowledge, attitudes and behavior. A clinical examination was performed to assess dental caries and gingival bleeding (only 12- to 15-year-olds). SPSS 17.0 statistical software was used to analyze the data.

**Results:**

The decayed, missing, filled teeth (DMFT) index scores of 12- to 15-year-olds and 4-to 6-year-olds averaged 0.45 and 3.05, respectively. The caries prevalence was 23.9% in 12- to 15-year-olds and 67% in 4-to 6-year-olds. Additionally, 45.2% of the 12- to 15-year-olds had gingival bleeding and 62.8% had calculus. The oral health knowledge of the subjects was generally poor, whereas they held very positive attitudes toward oral health. A low number of participants reported that they brushed their teeth at least twice daily. Moreover, a statistically significant relationship was found between oral health knowledge scores, tooth brushing frequency and DMFT scores as well as gingival bleeding in the 12- to 15-year-olds. Frequency of sweets consumption was strongly related to dmft scores in the 4- to 6-year-olds.

**Conclusion:**

The oral health status, oral health knowledge and behaviors among village children in Shaanxi Province are poor. Oral health education to improve oral health knowledge and to increase the frequency of tooth brushing should be undertaken in the rural schools in western China.

## Background

Oral disease is a major public health problem with a high prevalence and incidence, especially in low-income populations [1]. More than half of China’s population resides in rural areas with lower average incomes than urban areas [2]. In the third Chinese National Oral Health Survey, conducted in 2005, the dental caries prevalence among 5-year-old children was higher in rural areas (70.2%) than in urban areas (62%). In 2005, 28.6% of rural 12-year-olds had dental caries, a prevalence that had not declined significantly since the second Chinese National Oral Health Survey 10 years earlier [3, 4]. Moreover, although average income had increased with growth of the rural economy [5], the percentage of untreated carious teeth was also higher among rural children than urban children, with no obvious decrease since the previous survey. Additionally, gingival bleeding and calculus were prevalent conditions in rural China; calculus was present in 72.2% of 12-year-olds in western rural areas [3].

The economy of Shaanxi Province in China ranks in the middle to lower level in China. There is little systematic data available from this region to analyze oral health status among its inhabitants. An oral epidemiological survey conducted in selected areas in Shaanxi Province in 1992 and 1993 showed that the caries prevalence was 36% among rural 5-year-olds and 18% among rural 12-year-olds [6]. The third National Oral Health Survey (2005) reported that the prevalence of caries among rural 5-year-olds in Shaanxi Province was 58.5% [7]. This value was far from the oral health goal established in 1994 by the World Health Organization that 90% of 5- to 6-year-olds should be caries-free by 2010. The current dental caries prevalence and gingival health status of rural children in Shaanxi Province were unknown before the present survey.

Adequate oral health knowledge is essential to instill appropriate oral health behavior to prevent oral diseases [8–10]. Analysis of oral health knowledge, attitudes and behavior in a population allows us to determine the risk factors for oral diseases and to develop behavior modification strategies. However, information about the oral health knowledge, attitudes and behavior among rural children in Shaanxi Province has been scare. Therefore, the objectives of the present analysis were two-fold. Our first goal was to describe the oral health status, and oral health knowledge, attitudes and behavior among 4- to 6-year-olds and 12- to 15-year-olds in villages in Shaanxi Province, western China. Our second goal was to analyze the relationship between dental status and oral health knowledge, attitudes and behavior to identify the possible risk factors for dental caries and gingival bleeding in this population.

## Methods

### Sample size and selection of children

Multi-stage cluster sampling was used to select the sample in this study performed from March to May 2012, with assistance from the local health and education authority. The ethics committee of the School of Stomatology at Xi’an Jiaotong University approved this study.

The sample size was calculated based on the prevalence of dental caries reported by a recent survey in Yunnan Province, western China (73.6% among 5-year-olds and 53.5% among 12-year-olds) [11], an error margin of 10%, a 95% confidence interval and a design effect of 1.5. The estimated sample size was 207 for 4- to 6-year-olds and 501 for 12- to 15-year-olds.

Because the objectives of the present survey were to describe the oral health status and risk factors of children living in villages in Shaanxi Province, and because children attending the township schools all lived in the villages, the sample was selected from township schools. In 2012, there were 90 townships in Shaanxi Province. With reforms in the distribution of primary and middle schools in Shaanxi Province from 2006 to 2010, there are generally one or two primary schools and one middle school in each township. According to our pre-survey, each primary school has approximately 150–200 students and each middle school has approximately 200 students. Details of our sampling strategy are as follows. First, the three major counties of Shaanxi Province were chosen for the survey, mainly based on their geographic location. Second, one rural township was selected in each region with simple random cluster sampling. Third, all 4- to 6-year-olds in the key kindergarten and key primary schools of each township were recruited, and all 12- to 15-year-olds in key middle schools were selected.

### Questionnaire

The informed consent forms and structured questionnaires were provided to the 12- to 15-year-olds and to the caregivers of the 4- to 6-year-olds before the physical examination. The structured questionnaire, which collected information about the subject’s demographic background, oral health knowledge, attitudes and behavior, and perceived oral health conditions, had been used in the third National Oral Health Survey in China. If the children and their parents or caregivers agreed the study, the 12- to 15-year-olds and the caregivers of the 4- to 6-year-olds signed the informed consent. Then they were interviewed face-to-face by trained interviewers. To avoid potential information bias, interviewers explained the purposes and confidentiality of the survey and explained specifically that the study had no impact on participants’ examinations. Interviewers verified the completeness of questionnaires at collection.

### Clinical examination

All children underwent a clinical examination to assess dental caries and 12- to 15-year-olds also underwent evaluation of their periodontal condition. Randomly selected duplicate examinations of 10% of participants were performed to assess inter-rater reliability of two examiners with Cohen’s kappa statistic. The kappa value was 0.7 about dental caries and 0.8 about the gingival bleeding, indicating a better agreement in the two examiners. The World Health Organization’s 1997 [12] caries diagnostic criteria for decayed, missing and filled teeth (DMFT) were used to evaluate dental caries status. Gingival bleeding and calculus of all teeth were examined in participating 12- to 15-year-olds by trained dentists using the World Health Organization’s method [12] to evaluate periodontal condition (0 = no gingival bleeding, 1 = gingival bleeding, X = missing tooth; 0 = no calculus, 1 = calculus, X = missing tooth). Clinical examinations were performed under field conditions, with the use of plane mouth mirrors, CPI periodontal probes and portable clinic lights.

### Data analysis

Seven questions were posed to 12- to 15-year-old students concerning the causes and prevention of tooth decay and gum disease; the caregivers of 4- to 6-year-olds were asked three oral health questions, mainly on prevention of tooth decay. A "dental knowledge score" was calculated by adding the total number of items answered correctly by the subjects, excluding responses such as, "do not know". Thus, dental knowledge scores ranged from 0 to 7 for 12- to 15-year-olds and from 0 to 3 for 4- to 6-year-olds, with higher scores indicating better dental knowledge. Four items concerning the importance of oral health were included to explore subjects’ attitudes toward oral health. Dental attitude scores were calculated by counting the total number of statements to which the subjects showed a positive attitude. This score ranged from 0 to 4, with a higher score indicating a more positive attitude. Sweets consumption was scored in this study based on frequency of consumption of desserts, sugar/chocolate, sugar water, carbonated beverages/juice and sweetened milk. For each type of sweet, 1 point = rarely or never consumed, 2 points = less than four times per month, 3 points = once per week, 4 = more than once per week but less than seven per week, 5 = once per day, 6 = more than once a day. Scores ranged from 0 to 30, with higher scores indicating more frequent consumption of sweets.

All data were entered in Microsoft Access with double entry. SPSS 17.0 statistical software (SPSS, Inc., Chicago, IL, USA) was used to analyze the data. Simple frequency tables and descriptive statistics (means and standard deviations) were processed and analyzed by chi-square and Fisher’s exact tests. To assess the relative effect of oral health knowledge, attitudes and behavior on the presence of dental caries and gingival bleeding, the scores for these items were first analyzed with bivariate associations. Those items associated with dental caries or gingival bleeding were further analyzed with multiple linear regression and logistic regression analysis, respectively.

## Results

### Profile of children

The response rates were 95% (n = 424) for 4- to 6-year-olds and 100% (n = 564) for 12- to 15-year-olds. The percentage of male participants was 56.1% among 12-to 15-year-olds and 57.6% among 4- to 6-year-olds.

### Caries and periodontal condition

In this survey, the mean DMFT of 12- to 15-year-olds was 0.45 ± 1.08 (range, 0–8), and the mean dmft of 4- to 6-year-olds was 3.05 ± 0.36 (range, 0–18). The prevalence of dental caries in 12- to 15-year-olds and 4- to 6-year-olds were 23.9% and 67%, respectively, at the time of examination. Dental treatment, determined as the percentage of total caries treated, and calculated with the FT/DMFT (ft/dmft) index, averaged only 6.67% for 12- to 15-year-olds and 0.33% for 4- to 6-year-olds. The decay component, representing untreated dental disease and calculated with the DT/DMFT (dt/dmft) index, was 93.33% for 12- to 15-year-olds and 95.74% for 4- to 6-year-olds. Additionally, 45.2% of 12- to 15-year-olds had gingival bleeding and 62.8% had calculus. No significant difference in caries prevalence was found between boys and girls in either age group. Among 12- to 15-year-olds the prevalence of gingival bleeding and calculus was similar between boys and girls. The DMFT scores, their components, their indices and the percentage of children with gingival bleeding and calculus are shown in Table 1.

**Table 1:** **DMFT scores, components, indices and prevalence of gingival bleeding and calculus in each group**

	12- to 15-year-olds	4- to 6-year-olds
DT (dt)	0.42 ± 1.03	2.92 ± 0.32
MT (mt)	0	0.13 ± 0.07
FT (ft)	0.03 ± 0.19	0.01 ± 0.01
DMFT (dmft)	0.45 ± 1.08	3.05 ± 0.36
DT/DMFT (dt/dmft)	93.33%	95.74%
FT/DMFT (ft/dmft)	6.67%	0.33%
PP of dental caries	23.9%	67%
PP of gingival bleeding	45.2%	**–**
PP of calculus	62.8%	**–**

### Oral health knowledge

Among 12- to 15-year-olds, only 5.3% had accurate knowledge concerning dental plaque; 41.5% agreed that dental caries is mainly caused by bacteria. Sugar was identified as a cause of dental caries by 76.6% of respondents in this age range, whereas only 3.7% of students knew that fluoride toothpaste helps prevent dental caries. Gingival bleeding during tooth brushing was seen as normal by 27.1% of 12- to 15-year-olds, and 53.7% agreed that bacteria are one cause of gingivitis. Only 46.8% responded that tooth brushing is an important means of preventing gingival bleeding. The mean oral knowledge score was 2.40 ± 1.31; scores of boys and girls were similar. The indexes are shown in Table 2. Among 12- to 15-year-olds, 62.1% indicated that their oral health information was obtained from radio or television, while only 4.7% received information from posters in hospitals and 8% reported that oral health education in school was a source of information.

**Table 2:** **Oral health knowledge among 12- to 15-year-olds and caregivers of 4- to 6-year-olds**

	Agree	Disagree	Have no idea
12-to 15 year-olds (n = 564)			
Dental plaque is formed by colonizing bacteria trying to attach themselves to the tooth’s smooth surface.	5.3%	24.9%	69.8%
Dental caries mainly caused by the bacteria.	41.5%	14.9%	43.6%
Having sugars can lead to the dental caries.	76.6%	6.9%	16.5%
The fluoride tooth paste is good for preventing dental caries.	3.7%	2.6%	93.7%
Bacteria was one of the reasons causing the gingivality.	53.7%	8.5%	37.8%
Gingival bleeding is normal when tooth brushed.	27.1%	51.1%	21.8%
Tooth brushing was not an important ways to prevent gingival bleeding.	12.2%	46.8%	41%
4-to 6 year- olds (n = 424)			
Pit and Fissure Sealant could prevent the dental caries.	5.8%	4.2%	90%
The dental caries of deciduous teeth did not need to be treated.	5.3%	23.8%	70.9%
The fluoride tooth paste is good for preventing dental caries.	9.5%	1.6%	88.9%

Among the caregivers of 4- to 6-year-olds, only 5.8% answered that pit and fissure sealant could prevent dental caries and only 23.8% disagreed that dental caries in deciduous teeth do not require treatment. Moreover, only 9.5% knew that fluoride toothpaste helps prevent dental caries. The mean score of oral knowledge among caregivers was 1.30 ± 0.71. There was no difference between caretakers of boys and caretakers of girls. The indexes are shown in Table 2. Additionally, 70.8% of caregivers indicated that they received oral health information from radio or television.

### Oral health attitudes

Regular dental check-ups were recognized as a means of preventing dental problems by 73.4% of 12- to 15-year-olds; 86.2% indicated that the state of their teeth was very important to them, 85.1% disagreed that dental health is decided at birth and is not related to self-care and 88.3% indicated that self-care is important to prevent dental problems. The mean score of oral health attitude was 3.36 ± 0.98, with no difference between boys and girls. Some indexes of oral health attitudes are shown in Table 3.

**Table 3:** **Oral health attitudes of 12- to 15-year-olds and caregivers of 4- to 6-year-olds**

	Agree	Disagree
12-to 15 year-olds (n = 564)		
Regular dental check-up prevent dental problem.	73.4%	26.6%
The state of my teeth is great important to me.	86.2%	13.8%
State of teeth is decided at birth and is not relation to self-care.	14.9%	85.1%
Self-care is important for preventing dental problems.	88.3%	11.7%
4-to 6 year- olds (n = 424)		
The state of the teeth is greatly important to children.	98.8%	1.2%
State of teeth is decided at birth and is not relation to self-care.	17.5%	82.5%
Self-care is important for preventing dental problems.	97.6%	2.4%
Preventing dental caries of the first permanent molars was important for children.	80.9%	9.1%

Among caregivers of 4- to 6-year-olds, 98.8% answered that the state of children’s teeth is very important to children, 82.5% disagreed that dental health is decided at birth and is not related to self-care, 97.6% indicated that self-care is important to prevent dental problems and 80.9% agreed that preventing dental caries in the first permanent molars is important for children. The mean score of oral health attitude was 3.87 ± 1.01 among caregivers. There was no difference between caretakers of boys and caretakers of girls. Some indexes of oral health attitudes are shown in Table 3.

### Oral health behavior

Among the 12- to 15-year-old children, only 3.2% brushed their teeth at least twice daily and only 2.1% used fluoridated toothpaste. The mean score for frequency of sweets consumption was 14.21 ± 5.48. Approximately 41.5% of children in this age group had often or occasionally experienced toothache in the previous 12 months; 58.2% of these indicated that nothing was done for their toothache, while 16.5% reported that they took medication by themselves or had medication administered by their parents. Only 13.9% went to their dentist for toothache treatment. The percentage of students in this age range who had never seen a dentist during their lifetime was 51.6%. Toothache was the main reason for the students’ most recent dental visit and only 21.1% went to the dentist for regular dental check-ups. No participants had received preventive dental care. The main reason participants cited for not visiting a dentist was that their teeth were healthy (88%). Some indexes of oral health behavior are shown in Table 4.

**Table 4:** **Oral health behavior of 12- to 15-year-olds and 4- to 6-year-olds**

	12-to 15 year-olds (n = 564)	4-to 6 year- olds (n = 424)
*Frequency of tooth brushing*		
Brushing at least twice a day	3.2%	2.8%
Brushing once a day	44.1%	14.2%
Seldom or no brush	52.7%	83%
*Use of fluoridated toothpaste*		
Fluoridated	2.1%	2.1%
No- fluoridated	7.4%	14.2%
Have no idea	90.5%	83.7%
*Frequency of eating sweets*		
Two times or more per day	7.4%	42.7%
Once a day	12.7%	17.5%
Less than once a day	79.9%	29.8%
*Time since last visit to the dentist*		
Less than a year	9.6%	16.1%
1-2 years ago	23.9%	65.6%
3 or more years ago	66.5%	18.3%
*Reason for the last dental visit*		
Toothache	47.3%	30.8%
Check-up for the oral problem	21.1%	15.4%
Regular check-up	21.1%	15.4%
Tooth trauma	5.3%	0%
Getting preventive treatment	0%	7.7%
Others	5.2%	30.7%

Among caregivers of 4- to 6-year-olds, 2.8% reported that children brushed their teeth at least twice daily and 83% reported infrequent or no tooth brushing. Only 2.1% used fluoridated toothpaste. The mean score of sweets consumption frequency was 20.12 ± 4.9. Toothache was the main reason given for the most recent dental visit, and only 15.4% visited the dentist for regular dental check-ups. Preventive dental care had been performed in 7.7% of 4- to 6-year-olds. Healthy teeth was the main reason cited for not visiting a dentist (73.3%). Some indexes of oral health behavior are shown in Table 4.

### Possible risk factors for caries and poor periodontal condition

In our bivariate analyses, a statistically significant relationship was found between oral health knowledge scores and DMFT as well as gingival bleeding among 12- to 15-year-olds. However, the same relationship was not found among 4- to 6-year-olds. No relationship was found between oral health attitude score and the DMFT in either group. Sweets consumption scores were strongly related to dmft scores in the 4- to 6-year-olds, but not in the 12- to 15-year-olds. Tooth brushing frequency had a significant relationship with the DMFT score and gingival bleeding in 12- to 15-year-olds. Indexes are shown in Table 5.

**Table 5:** **Association between oral health knowledge, attitudes and behavior and mean DMFT scores and components**

	D	M	F	DMFT	Gingival bleeding
12-to 15 year-olds (n = 564)					
*Oral health knowledge score*	-0.257^**^	-	-0.173^*^	-0.215^**^	-0.241^**^
*Tooth brushing frequency*	-0.303^**^	-	0.126	-0.296^**^	-0.318^**^
*Time since last visit to the dentist*	-0.045^*^	-	-0.176^*^	-0.066	-
4-to 6 year- olds (n = 424)					
*Oral health knowledge score*	-0.190	-0.352^*^	-0.352^*^	-0.259	-
*Sweets consuming score*	0.460^**^	0.096	0.145	0.441^**^	-
*Time since last visit to the dentist*	-0.218^*^	-0.269^**^	-0.224^*^	-0.254^*^	-

^*^p < 0.05; ^**^p < 0.01.

Regression model analysis showed that the 12- to 15-year-olds with higher oral health knowledge scores and more frequent tooth brushing were less likely to have dental caries, while the 4- to 6-year-olds who ate sweets more frequently had more dental caries. Moreover, the 12- to 15-year-olds with lower frequency of tooth brushing were more likely to have gingival bleeding. Indexes are shown in Table 6.

**Table 6:** **Regression models analysis of dental caries (DMFT or dmft) among children in rural Shaanxi Province**

Independent values	Coefficient (DMFT or dmft)	p
12-to 15 year-olds (n = 564)		
*Oral health knowledge score*	-0.186	0.013
*Tooth brushing frequency*	-0.245	0.001
*Time since last visit to the dentist*	-0.06	0.411
df = 3; p <0.01; r^2^ = 0.110.		
4-to 6 year- olds (n = 424)		
*Sweets consuming score*	0.437	0.000
*Oral health knowledge score*	-0.136	0.148
*Time since last visit to the dentist*	-0.038	0.175

df = 3; p <0.01; r^2^ = 0.209.

## Discussion

There is little systematic data available to analyze oral health status among village children in China. In the present study, the oral health status and related risks of children in this region were analyzed. Although this is a regional survey with a limited sample, it does provide insights into the oral health status and oral health knowledge, attitudes and behavior among rural children in western China. In the present study, the caries prevalence among 4- to 6-year-olds and 12- to 15-year-olds in the villages of Shaanxi Province were 67% and 23.9%, respectively. These values are similar to those reported by the third Chinese national survey among rural 5-year-olds and 12-year-olds in western China [3]. The caries prevalence among 4- to 6-year-olds in the present survey was higher than that reported among 5-year-olds in developed countries, such as Norway [13]. Moreover, caries prevalence in this population remains far from the oral health goal set in 1994 by the World Health Organization, that 90% of 5- to 6-year-olds should be caries-free by 2010. Additionally, dominant dt/DT-components were found both in rural 4- to 6-year-olds and in 12- to 15-year-olds in this study. The findings indicate that caries prevalence was relatively high among village children in Shanxi Province and that dental caries did not usually receive treatment, similar to the situation in most developing countries [14–16]. The periodontal health condition of the 12- to 15-year-old rural children surveyed was generally poor, but it was slightly better than that reported among 12-year-olds in Laos [15].

In general, oral health knowledge among 12- to 15-year-olds and among caregivers of 4- to 6-year-olds in the rural areas of Shaanxi Province was poor and was worse than that in southern China [17, 18]. For example, only 28.3% of caregivers of 4- to 6-year-olds disagreed with the statement that dental caries in deciduous teeth did not require treatment, whereas the corresponding percentage in southern China was 51.6% [18]. Lack of knowledge about fluoride toothpaste was found in 93.7% of 12- to 15-year-olds, a percentage higher than that among school children in Nepal [16]. Additionally, oral health information was primarily received from television and radio, a finding similar to that among adults of Guangdong Province in southern China [17]. Although oral health education programs such as the annual Love Teeth Day campaign to encourage the implementation of oral health education have been conducted since 1989 [19], education at school was indicated as a source of oral health information by only 5% of 12- to 15-year-olds in the present survey. This low percentage indicates that oral health education programs are less common in rural areas in western China and that they should be enhanced in these areas. Although Shaanxi remains a relatively poor province in China, with the continuous economic growth of the last 20 years, television was present in 100% of the rural participants’ homes in this study, and television is a primary means of disseminating oral health messages to the rural population. Thus, more oral health education could be conducted via television to improve oral health knowledge among village children. Despite their poorer dental knowledge, the subjects in this study held very positive attitudes toward oral health, similar to those found among children and adults in Guangdong Province in southern China [17], a finding that might be partly explained by over-reporting.

Few participants in this survey reported tooth brushing at least twice daily and very few were using fluoridated toothpaste, percentages worse than those found in central and eastern China [3] and in other developing [15, 16, 20] and developed countries [21]. These findings may result from poor oral health knowledge in this region. Toothache was the most common reason for the most recent dental visit, and preventive dental care was very uncommon. These results indicate that rural children lack awareness of how to prevent dental disease.

To assess the possible risk factors for dental caries and gingival bleeding, oral health knowledge, attitude and behaviors were evaluated with bivariate associations and multiple regression analysis. Many studies have reported an association between oral health knowledge scores and oral health status [8, 9, 17], an association confirmed among 12- to 15-year-olds in the present study. This association suggests that oral health knowledge should be enhanced via increased oral health education among village children to improve their oral health status. However, the oral health knowledge of the caregivers of 4-to 6 year-olds was not associated with dental caries among the children, a result not consistent with that of another survey [22]. Additionally, higher frequency of sweets consumption was associated with higher dmft among the 4-to 6 year-olds in this survey. Previous studies have found a similar correlation [23, 24], suggesting that preschool oral health education should focus on the consumption of sugars to more effectively prevent deciduous dental caries. However, no significant association between sweets consumption and DMFT scores was found among 12-to 15-year-olds, a finding similar to that reported in a previous study [15]. Frequent tooth brushing was strongly associated with lower DMFT scores and less gingival bleeding in 12- to 15-year-olds in the present study, whereas no significant association was found among 4-to 6-year-olds. This difference might be explained by the fact that most of the 4- to 6-year-olds seldom brushed their teeth, suggesting that tooth brushing should be encouraged in rural areas. Additionally, high caries values were found to be associated with a recent dental visit among village children in Shaanxi province. This finding agreed with that of a survey in Laos, and somewhat indicated that dental visits are often prompted by pain and discomfort rather than by the need for regular preventive care [15].

## Conclusion

Dental caries prevalence among 4- to 6-year-old village children in Shaanxi Province, western China, was relatively high, while the prevalence of dental caries among 12- to 15-year-olds was low, although periodontal condition was poor in that age group. Moreover, children lacked knowledge about dental caries, gum disease and the use of fluoride. A strong association was found between sweets consumption and the presence of dental caries among 4- to 6 year-old village children. Preschool oral health education should emphasize reduced consumption of sugars in the rural areas of Shaanxi Province. Tooth brushing and oral health knowledge were inversely associated with dental caries and gingival bleeding in 12- to 15-year-olds. More oral health education programs should be organized in rural schools to improve oral health knowledge and the frequency of tooth brushing in this population.
